# Current Concepts in the Biochemical Mechanisms of Glaucomatous Neurodegeneration

**DOI:** 10.5005/jp-journals-10008-1137

**Published:** 2013-05-09

**Authors:** Syed Shoeb Ahmad, Shuaibah Abdul Ghani, Theodora Hemalata Rajagopal

**Affiliations:** Ophthalmologist, Department of Ophthalmology, Queen Elizabeth, Hospital Kota Kinabalu, Malaysia; Ophthalmologist, Department of Ophthalmology, Queen Elizabeth, Hospital Kota Kinabalu, Malaysia; Ophthalmologist, Department of Ophthalmology, Queen Elizabeth, Hospital Kota Kinabalu, Malaysia

**Keywords:** Glaucoma, Biochemical, Neural, Degeneration.

## Abstract

Glaucoma is now regarded as a neurodegenerative disorder. A number of theories including the mechanical and vascular models have been used to explain the pathogenesis of glaucoma. However, there is now increasing evidence of biochemical molecules which may play a part in it's causation. These biochemical mechanisms include the role of excitatory aminoacids, caspases, protein kinases, oxygen free radicals, nitric oxide, TNF-alpha, neurotrophins and metalloproteins. This paper reviews these new developments which form the biochemical basis of glaucomatous neural degeneration.

**How to cite this article:** Ahmad SS, Ghani SA, Rajagopal TH. Current Concepts in the Biochemical Mechanisms of Glaucomatous Neurodegeneration. J Current Glau Prac 2013;7(2):49-53.

## INTRODUCTION

Glaucoma is now regarded as a neurodegenerative disease entity.^1,2^ A characteristic feature of glaucoma is the loss of retinal ganglion cells (RGCs).^3-5^ The death of RGCs is initiated when a pathological event, such as ischemia, axonal injury or changes in the lamina cribrosa lead to activation of apoptosis. Historically, glaucoma causation has been explained on the basis of mechanical and vascular theories. However, over the past few years a number of biochemical molecules have been implicated in the ability to launch apoptosis. This review takes a look at these biochemical mechanisms responsible for glaucomatous degenerative changes and which may prove to be the new frontier in glaucoma management.

## GLUTAMATE EXCITOTOXIC HYPOTHESIS

The major excitatory amino acids of the central nervous system *viz* glutamate, aspartate and others are implicated in the common final pathway of neural demise.^1-5^ Endothelin-1–induced ischemia of the optic nerve is associated with a statistically significant elevation in the vitreous concentrations of glutamate (264 *vs* 41%), aspartate (269 *vs* 31%) and glycine (232 *vs* 26%) compared to controls.^6^ In another study intravitreal levels of glutamate were found to be elevated in glaucoma patients (27 *vs* 11 mM).^7^ The excessive levels of these excitotoxins are deemed to be responsible for neuronal swelling, lysis and death.

The ‘glutamate excitotoxic hypothesis' was put forward to explain the mechanism of ischemic injury.^7^ This school of thought maintains that the lack of oxygen itself is not sufficient to cause damage to ischemic tissue. Instead, the release and receptor binding of glutamate makes the subsequent damage more likely. Glutamate transporters (excitatory amino acid transporter or EAAT) or molecules, which ordinarily regulate extracellular glutamate, have also been implicated in raised levels of glutamate.^8^ Failure of these transporters leads to elevated glutamate, which can cause alterations in glutamate receptor expression. Glutamate is also closely related to and acts through N-methyl-D-aspartate (NMDA) receptors.

## NMDA AND GLUTAMATE BINDING

The NMDA receptor is a ligand-gated ion channel. These channels are transmembrane ion channels which open or close in response to the binding of a chemical messenger (i.e. a ‘ligand'), which could be in the form of a neurotransmitter. The NMDA receptor has two binding sites: One for NMDA or glutamate and the other for glycine. Mg^++^ (a physiological inhibitor of NMDA receptor activation) from the receptor site is also required. When the nerve is depolarized, Mg^++^ is removed from the receptor. The overstimulation of the NMDA receptor by the high levels of glutamate leads to an increased influx of calcium into the neuronal cell, leading to toxicity and triggering apoptosis of RGCs. Studies have shown that both competitive and noncompetitive NMDA antagonists enhance functional recovery in hypoxic tissue, directly reduce neuronal vulnerability to hypoxic insults and are capable of reducing hypoxic damage. However, prolonged NMDA receptor blocking, as required in chronic conditions like glaucoma, is not feasible. It can lead to seizures, psychosis, coma and even death. The use of noncompetitive antagonists to protect against excessive levels of glutamate might be a safer method to prevent the adverse effects of prolonged receptor blockade. The noncompetitive antagonist memantine is neuroprotective in several models of RGC excitotoxicity.^9^

## EXCITOTOXIC NEURAL DEGENERATION

Excitotoxicity refers to the clinical condition in which amino acids excite the nerve excessively, resulting in neurotoxicity and neuronal death.^10^ Therefore, excitotoxicity refers to the dual action of these amino acids in which neuronal excitation occurs in normal circumstances and cell toxicity occurs when they are present in excess. Following neuronal injury, excitatory amino acids are released into the surrounding medium. The released amino acids, specifically glutamate, activate two kinds of receptors: (i) Ionotropic and (ii) metabotropic.

The preferred agonists of ionotropic receptors are NMDA, alpha-amino-3-hydroxyl-5-methlyl-4-isoxandepro-pionic acid (AMPA) and kainite (KA). The metabotropic receptors are linked to G-regulatory protein.

Acute phase reactions, which take place following glutamate release, are:

1. Na^+^ enters the cell primarily via AMPA receptor channels.
2. Cl^–^ and water passively follow Na^+^ resulting in cellular swelling.

However, the cellular swelling is rarely fatal and the cell may recover from the insult.

Delayed phase reactions in neuronal injury are:

1. Ca^++^ enters the cell primarily through NMDA channels.
2. Ca^++^ influx also occurs indirectly through non-NMDA receptors.
3. Depolarization leads to Ca^++^ influx through voltage-sensitive calcium channels (VSCC).

These reactions lead to altered calcium homeostasis and induce a cascade of metabolic reactions.

Increased cytoplasmic Ca^++^ can activate a number of calcium-dependent enzymes including protein kinase C (PKC), phospholipase A2, phospholipase C, Ca/calmodulin-dependent protein kinase II, nitric oxide synthase (NOS) and various protease and lipase leading to the formation of free fatty acids and destruction of membrane stability. Phospholipase activation causes cell membrane breakdown liberating phospholipase A2. This triggers arachidonic acid and free radical formation. Phospholipase A2 also liberates endonuclease which breaks the DNA genome. The increase in intracellular calcium causes accumulation of calcium in mitochondria, which disturbs the process of oxidative phosphorylation. This leads to decreased ATP synthesis. It also leads to anaerobic metabolism of glucose causing lactose accumulation. The lactose accumulation, in turn, causes cellular acidosis. This disturbs the metabolic functions and decreases the buffering capacity of the cell, ultimately causing cellular death.

Glutamate also activates metabotropic receptors. This stimulation activates protein G, which in turn, activates phospholipase C. This leads to hydrolysis of phosphati-dylinositol 4,5-bisphosphate. This liberates the dual messengers inositol 1, 4, 5-trisphosphate (IP3) and diacylglycerol (DAG). IP3 causes Ca^++^ release from the endoplasmic reticulum leading to increased intracellular Ca^++^ levels. DAG, in the presence of Ca^++^, activates PKC, calmodulin and calmodulin kinase II. PKC has two main actions: One, it alters the phosphorylation of proteins which are integral parts of receptor and ion channels. This disturbs the cellular functions. Secondly, PKC induces Ca^++^-dependent glutamate release and Ca^++^ entry through the VSCCs.

The synaptic vesicles of nerve terminals contain glutamate. Depolarization of the nerve terminal releases glutamate into the synaptic cleft which generates its effects through specific glutamate receptors. Carriers in neurons and glial cells are responsible for removal of glutamate from the synaptic cleft. Under normal conditions this removal occurs efficiently. This timely glutamate removal from the nerve terminal stops glutamate's effects on neurons. The glutamate which is carried to the glial cells or neurons by the carriers is recycled and again concentrated in synaptic vesicles.

Excessive stimulation of the nerves alters the Ca^++^ homeostasis and leads to excitotoxicity. The mechanisms of excitotoxicity include hypersensitivity of postsynaptic neurons to glutamate, insufficient removal of glutamate or abnormality in glutamate receptors. There might be an abnormally high Ca^++^ conductance of glutamate receptors in some disease states. In hypoxic and ischemic states, large increases in extracellular glutamate and marked depression of glutamate uptake system are found to occur.

Excitotoxic cell death is usually considered a Ca^++^– dependent process. However, in certain neuronal systems excitotoxic cell death is independent of Ca^++^. There is strong evidence to suggest the role of extracellular Cl^–^ in this process.^11^ The lethal Cl^–^ entry occurs through GABA and glycine receptors. Studies have shown that GABA receptor blockade provides complete neuroprotection.

## CASPASES

Neuronal apoptosis is also mediated by a family of aspartate-specific cysteine proteases known as caspases. The term caspases was coined to denote the **C**ysteine requiring **ASP**artate prote**ASE** activity of these enzymes. Caspases are enzymes involved in the destruction of cells.^12^ The caspases exist as immature procaspases and need to be cleaved to become active. These proteins breakdown key cellular substrates required for normal cellular function. Study of apoptosis after ischemia-reperfusion injury has shown that caspases may have a pivotal role in the early events of the apoptotic pathway(s). Caspases can be divided into two main classes: (i) Initiator and (ii) effector caspases. Initiator caspases (e.g. caspase-9) are the upstream activators of the effector caspases (e.g. caspase-3). Effector caspases are the so-called ‘executioners' in the cell.

Cytochrome C (cyt C) and ATP (or dATP) binds to Apaf-1 leading to its activation. Apaf-1 can then oligomerize, which by binding to the prodomains of procaspase-9, can bring procaspase-9 proteins in close proximity to one another. As a result, the procaspase-9 cleave each other leading to the formation of mature caspases-9 tetramers. The mature caspases then go on to cleave and activate other effector caspases (e.g. caspases-6 and -2) or other cellular substrates. Caspase-6 can cleave and activate other effector caspases (e.g. procaspase-8 and -10). All of these effector caspases cleave and activate other effector caspases.^13^ They can also cleave other cellular substrates like caspase-activated deoxyribonuclease (CAD) and ICAD (inhibitor of CAD). CAD cleaves the DNA into fragments, forming the characteristic DNA laddering of apoptotic cells. The effector caspases can also cleave structural proteins such as nuclear lamins, which maintain the integrity of the nucleus. When cleaved by effector caspases, the nucleus undergoes condensation, a typical feature of apoptosis.

## JNK PATHWAY

The c-JUN N-terminal protein kinase (JNK) or stress-activated protein kinase (SAPK) pathway is one of several mitogen-activated protein kinase (MAPK) pathways. MAPKs are important mediators for intracellular signalling in cells. The transcriptional activities of many transcription factors are regulated by JNK. In particular, regulation of c-JUN by JNK modulates stress-induced apoptosis. It is involved in controlling a wide range of biological processes including proliferation, inflammation, apoptosis and morphogenesis. JNK is activated by diverse stimuli including environmental stress, proinflammatory cytokines and mitogenic stimuli in mammalian cells. The regulation of JNK pathway by its regulators in conjunction with other signalling pathways may allow JNK to regulate a variety of cellular functions. Retinal ischemia-reperfusion injury leads to expression of c-JUN causing apoptosis of the RGCs.

Glutamate toxicity may also involve the JNK group of MAPKs. One member of the JNK family, JNK 3, may be required for the stress-induced neuronal apoptosis, as it is selectively expressed in the CNS. Excitotoxicity-induced apoptosis is not found in the hippocampus of mice lacking the JNK 3 gene.^14^ Disruption of the gene encoding JNK 3 in mice caused them to be resistant to the excitotoxic glutamate-receptor Kainic acid. Neuroprotection is afforded due to the extinction of a JNK 3–mediated signalling pathway which is an important component in the pathogenesis of glutamate neurotoxicity.

## OXYGEN FREE RADICALS

Oxygen free radicals (OFRs) have also been implicated in RGC death.^15^ OFRs are molecules with one or more unpaired electrons. Normal cellular metabolism constantly produces OFRs. However, antioxidant enzymes like superoxide dismutase, catalase and glutathione peroxidise remove them, as also free radical scavengers like glutathione, alpha-tocopherol (vitamin E) and beta-carotene. OFRs react with lipids, nucleic acids and proteins. They are responsible for tissue injury during ischemia and in secondary degeneration following reoxygenation (reperfusion), once the ischemic injury has ceased.

Intracellular calcium is also responsible for free radical formation. Increased calcium level on one hand activates phospholipases, leading to arachidonic acid oxidation and free radical formation. On the other hand it also activates xanthine dehydrogenase to xanthine oxidase, which produces uric acid and superoxide radical. Xanthine oxidase is a rich enzymatic source of free radicals. Increased intracellular calcium and exposure to glutamate also decreases cysteine uptake. Cysteine is a precursor of glutathione, which is important for the removal of free radicals. Free radical accumulation in the cell activates nitric acid synthase which leads to nitric oxide (NO) production. High levels of certain reactive species are formed through generation of hydroxyl radical (as a result of the Fenton reaction) or peroxynitrite (resulting from the reaction of NO and superoxide anion).

In brain trauma and ischemia OFRs are formed due to extensive oxidation of proteins and lipid peroxidation. A dysfunction in the RGCs endogenous superoxide/peroxide scavenging systems has also been found. Mice over-expressing Cu-Zn-superoxide dismutase (Cu-Zn-SOD) are shown to have less number of surviving RGCs following optic nerve crush injury,^16^ compared to normal mice. Findings of retinal neuron culture studies show that the presence of lipid peroxidation inhibitors or a decrease in oxygen availability inhibits RGC death.

## NITRIC OXIDE SYNTHASE-2

NO is a free radical gas. NO has been identified in the RGCs, amacrine cells of the inner nuclear layer of the retina and in the blood vessels of the choroid and the limbus. In the CNS it acts as a neurotransmitter. It has been found to have both neurodestructive and neuroprotective properties. Elevated hydrostatic pressure induces astrocytes in the optic nerve head to express NOS-2.^17^ The excessive NO production, which is associated with NOS-2 may contribute to the neurotoxicity of RGCs in eyes with chronic moderately elevated intraocular pressure.^18^ NO is produced in response to activation of the NMDA subtype of glutamate receptor. When glutamate activates NMDA, Ca^++^ channels open and Ca^++^ influx occurs. High levels of intracellular Ca^++^, along with intracellular calmodulin and NADPH stimulate the NOS enzyme to produce NO from arginine. NO is not stored in synaptic vesicles and diffuses out. In the presence of large, toxic levels of glutamate, NOS neurons behave like macrophages releasing large amounts of NO to kill nearby neurons. As a free radical, NO can damage DNA and result in mutations. The DNA strand breaks activate polyadenosine diphosphate (ADP)-ribose synthetase (PARS). This enzyme is important for DNA repair, cellular differentiation, transformation and gene arrangements. In turn PARS catalyses poly-ADP ribosylation of many nuclear proteins. With overstimulation of PARS in stroke or metabolic stress, NAD, which is a substrate for PARS is depleted. The destruction of DNA overwhelms its repair and the neuron dies. NO also inhibits ribonucleotide reductase, which is necessary for DNA synthesis and repair. Therefore, DNA repair is delayed in the presence of NO. It is also noted that DNA fragments prolong the activation of PARS. Excessive NO, released by reactive astrocytes leads to the formation of peroxynitrite, which damages the axons of the RGCs at the level of the lamina cribrosa.

NO is an important substance for transcellular signal transduction. Some reports show that neurons possessing NOS activity are actually more resistant to neuronal damage in ischemia.^19^ One mechanism proposed for the dual activity of NOS is that the chemical pathways involving distinct redox-related congeners of NO may either trigger neurotoxic or neuroprotective pathways. NO is found in different redox-related states: Nitrosonium ion (NO^+^), nitric oxide (NO^0^) and nitrosylation (NO^–^), which may explain its diverse activities. NO is also involved in the S-nitrosylation of some proteins, including the NMDA receptor. Nipradiol, a beta-blocker with NO donative action was also found to suppress NMDA-induced retinal damage in rats.^20^

## TNF-ALPHA

Tumor necrosis factor (TNF)-alpha is a cytokine produced by glia in human glaucomatous optic nerve head (ONH). It mediates a range of cellular responses, which have potentially detrimental consequences affecting multiple cell types. The expression of TNF-alpha and its receptor-1 (TNF-R1) have been studied in glaucomatous ONHs using double labeling fluorescence immunohistochemistry. Normal tissue shows constitutive expression of TNF-R1 in the vasculature of the ONHs but no positive labeling for TNF-alpha. In the glaucomatous ONHs expression of both TNF-alpha and TNF-R1 is upregulated, primarily in glial fibrillary acidic protein (GFAP)-positive astrocytes and appears to parallel the progression of optic nerve degeneration.^21^ Astrocytes constitutively express TNF-R1 and TNF-alpha stimulation has been found to induce expression of NOS-2. Thus, TNF-alpha contributes to the progression of optic nerve degeneration in glaucoma by both a direct effect on the axons of the RGCs and by inducing NOS-2 in astrocytes.

## NEUROTROPHINS

Neurotrophins are molecules essential for neuronal survival, development, function and in experimental models, regeneration of neurons. Initially thought to be derived from the brain, there is evidence of neurotrophin production locally in the retina also.^22^

Neurotrophic molecules are found to travel from the lateral geniculate body to the RGCs by retrograde axonal transport. Thus a normal axonal transport is essential for the health, support and survival of the RGCs. One of the NT molecules, found to have deficient supply to the retina in animal models of glaucoma is brain-derived neurotrophic factor (BDNF).^23,24^ This is a neurotrophic factor found in the brain and periphery including the kidneys and the prostrate. When the target neurons are deprived of retrograde neurotrophic support they undergo apoptotic death. The retrograde transport of BDNF is found to be obstructed when IOP is acutely elevated. The loss of BDNF to the RGCs results in NT support deprivation. This results in apoptotic death of the RGCs. Application of exogenous BDNF to the superior colliculus reduces RGC death. *In vitro* application of NTs, especially BDNF to isolated RGCs prolongs their survival. *In vivo* RGC survival is also found to be prolonged by intravitreal injection of BDNF in cases of RGC injury.^25^

## MATRIX METALLOPROTEINASES

The regulation and maintenance of aqueous humor outflow is dependent on a constant trabecular meshwork (TM) matrix turnover. This TM matrix turnover is mediated by a family of endopeptidase enzymes known as matrix metallo-proteinases (MMPs).^26^ The normal tissue homeostasis requires a balanced interaction between MMPs and tissue inhibitors of MMPs (TIMPs). Normally, the ratio of enzyme to inhibitor is 1:1. A disturbance in this ratio results in excessive or insufficient matrix degradation and matrix accumulation.

Decreased aqueous levels of endogenous MMP-2 activity also contribute to the abnormal matrix accumulation in the juxtacanalicular meshwork in patients with POAG.^27^ In the TM of patients with POAG there is an accumulation of abnormal extracellular matrix (ECM) in the form of a plaque. This leads to increased outflow resistance and chronic elevation of IOP. MMP-9 is a prerequisite for RGC apoptosis also. In MMP-9 knockout mice, as well as animals in which MMP-9 is pharmacologically inhibited, the RGC do not die by apoptosis. MMP-9 is also required for tissue remodeling of the ONH and contributes to the change of the shape of lamina cribrosa. MMP-9 is also found to play a role in the blood-brain barrier breakdown of the ONH which may lead to appearance of splinter hemorrhages.^28^

## CONCLUSION

Glaucoma management is reaching an interesting phase with new mechanisms being found. How many would pass the test of time and which will fall with more experience remains to be seen. The biochemical mechanisms point to only one direction of thought. There are many other mechanisms being investigated as well. It is possible that different mechanisms are active in different patients leading to glaucomatous degeneration.
